# Accuracy and image quality of wide-detector revolution CT angiography combined with prospective ECG-triggered CT angiography in the diagnosis of congenital aortic arch anomalies in Chinese children

**DOI:** 10.3389/fped.2022.1017428

**Published:** 2022-12-02

**Authors:** Hui-Jun Xiao, A-Lai Zhan, Qing-Wen Huang, Rui-Gang Huang, Wei-Hua Lin

**Affiliations:** Department of Radiology, Zhangzhou Affiliated Hospital of Fujian Medical University, Zhangzhou, China

**Keywords:** computed tomography angiography, congenital aortic arch anomalies, diagnosis, diadnosis, children

## Abstract

**Objective:**

To explore the accuracy and image quality of wide-detector revolution CT angiography combined with prospective ECG-triggered CT angiography in the diagnosis of congenital aortic arch anomalies in Chinese children.

**Methods:**

From January 2020 to July 2022, the clinical data of 57 children with congenital aortic arch anomalies confirmed by surgery were collected. All patients underwent CT angiography (CTA) with Revolution CT and transthoracic echocardiography (TTE) before the operation. The accuracy of CTA and TTE in the diagnosis of aortic arch anomalies was compared with the surgical results.

**Result:**

The diagnostic sensitivity, specificity, accuracy, positive predictive value, and negative predictive value of CTA and TTE for congenital aortic arch anomalies (including intracardiac and extracardiac structural abnormalities) were 92.2% and 85.5%, 99.4%, and 99.1%, 97.4% and 95.6%, 98.1% and 96.9%, and 97.2% and 95.2%, respectively. Regarding extracardiac structural malformations, the sensitivity of CTA was 100%, whereas that of TTE was 78.6% (*P* < 0.001). Regarding intracardiac structural malformations, the sensitivity of CTA was 84.5%, whereas that of TTE was 92.5% (*P* < 0.001). Regarding satisfaction with the images in aortic arch anomalies, surgeons noted that the CTA images were more useful for diagnosis and operation planning compared with TTE.

**Conclusion:**

Wide-detector revolution CT angiography combined with prospective ECG triggering can be routinely used to assess congenital aortic arch anomalies, providing adequate image quality and high diagnostic accuracy. However, limitations in the identification of intracardiac structural abnormalities are noted.

## Background

Congenital aortic arch anomalies can occur alone or in conjunction with other intracardiac defects. These anomalies are classified into three types: (1) aortic arch obstruction, including coarctation of the aorta (CoA) and interruption of the aortic arch (IAA); (2) abnormal aortic arch formation, including the right aortic arch, vascular ring, and cervical aortic arch; and (3) abnormal connection between the aorta and pulmonary artery, including different types of patent ductus arteriosus (PDA) and aortopulmonary window (APW) (1). Transthoracic echocardiography (TTE) is an effective method for the diagnosis of congenital heart disease (CHD), which can comprehensively reveal cardiac structural changes and hemodynamic abnormalities. However, the diagnosis of aortic arch malformations by TTE has certain limitations, and it is difficult to completely diagnose distal vascular disease and patients with poor sound transmission conditions (2). In recent years, CT angiography (CTA) has been widely used in the diagnosis of cardiovascular diseases. New technologies, such as the 16 cm *Z*-axis coverage, the 3D honeycomb collimator, and the volume HD wide-body reconstruction algorithm, allow the Revolution CT system to scan large areas in an axial manner, which not only reduces the radiation dose but also obtains high-definition image quality (3). Literature on aortic arch anomalies is relatively limited. Using surgical results as the gold standard, this paper aims to explore the accuracy and image quality of wide-detector revolution CT angiography combined with prospective ECG triggering in the diagnosis of congenital aortic arch anomalies in Chinese children.

## Patients and methods

### Patient population

This was a retrospective study. This study included 57 children with aortic arch anomalies confirmed by surgery between January 2020 and July 2022. Both CTA and TTE were required before surgery for the diagnosis of aortic arch anomalies.

### Examination of TTE

A GE-730 Voluson color Doppler ultrasound diagnostic instrument, S8 probe, probe frequency 3–8 mHz was used. The patients were placed in the supine or left lateral position, and the suprasternal fossa and parasternal, apical, and subxiphoid multisection and multiacoustic windows were used for scanning. The superior sternal fossa section was used to observe the course of the aortic arch as well as the origin, inner diameter, and length of each major branch vessel. The long axis of the left ventricle, the four chambers of the apex, the short axis of the great artery, and the upper sternum were observed. The size and range of motion of each chamber were measured routinely.

### Examination of CTA

The GE Revolution 512-slice multislice CT (MSCT) scanner was used in this study, and the scanning range was from the thoracic entrance to the lung floor. The scanning parameters were as follows: voltage 70 KV, current 200 mA, field of view 320 mm, matrix 512 × 512, collimator width 16 cm, tube rotation time 0.28 s/turn. The contrast medium was iopromide (300 mg I/ml), and the total amount was 1.5 ml/kg. The contrast medium was injected with a high-pressure syringe at 1.0–2.0 ml/s through the dorsal vein of the hand or the dorsum of the foot. After the injection, 10 ml of normal saline was used to flush the tube. Smartprep software was used to observe the peak concentration of contrast media and manually trigger the scan. After scanning, the original data were uploaded, and the image was reconstructed using a novel 70% Adaptive Statistical Iterative Reconstruction Veo (ASIR-V). The cardiac motion correction algorithm (Snapshot Freeze, SSF, GE Healthcare) was applied in the reconstruction to further improve the temporal resolution. The reconstructed image was uploaded to the Advantage Workstation AW 4.6 Image Workstation for postprocessing, including volume rendering (VR) and curved planar reconstruction (CPR), maximum intensity projection (MIP), and other postprocessing reconstructions. For children who could not cooperate with the examination, oral 10% chloral hydrate (0.2–0.3 ml/kg) or intramuscular injection of phenobarbital (5 mg/kg) was used for sedation.

### Image quality assessment

Subjective evaluation: A questionnaire survey was conducted by the chief surgeon to evaluate the images of cardiac structures and extracardiac great vessel structures. The questionnaire included four questions, which were divided into two parts. Part one, including questions 1 and 2, was conducted before the operation, and part two, including questions 3 and 4, was given after the operation. Questions 1 and 2 asked how helpful CTA and TTE were for preoperative diagnosis. Questions 3 and 4 assessed the accuracy of CTA and TTE. The answer to question 1 was graded on a 4-point scale ranging from unclear to very clear, with 1 being unevaluable and 4 being very clear. Question 2 reflected the surgeon's assessment of the helpfulness of CTA and TTE before the operation in terms of surgical methods and understanding of the patient's anatomy. Question 3 assessed the accuracy of the CTA and TTE measurements. Question 4 gauged the surgeon's approval of CTA and TTE as a useful tool in surgical assistance.

Objective evaluation: Two senior cardiac imaging diagnostic physicians placed the area of interest (ROI) at the position where the contrast agent entered the vena cava, the center of the main pulmonary artery, the ascending aorta, and the descending aorta. The area of the ROI was 2/3 of the vascular lumen. The corresponding CT value and SD value were measured, and the signal-to-noise ratio (SNR) was calculated as follows: SNR = CT/SD value.

## Results

**Figure 1:**
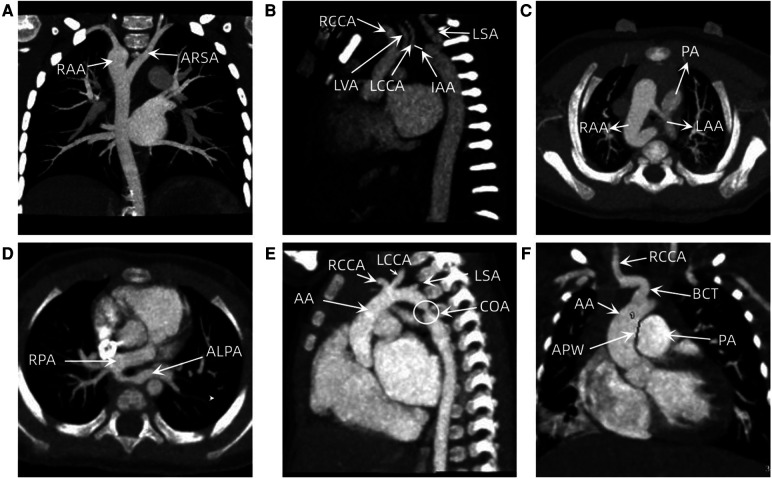
(**A**) A image of a six-year-old boy with the right aortic arch with ARSA. (**B**) A 17-day old boy with IAA. (**C**) A image of a 6-month-old child with double aortic arch and tracheomalacia. (**D**) A 9-month-old boy with a pulmonary artery sling and tracheal stenosis. (**E**) A images of a two-month-old boy with CoA. (**F**) A 27-day old boy with APW. RAA, right aortic arch; ARSA, aberrant right subclavian artery; RCCA, right common carotid artery; LVA, left vertebra artery; LCCA, left common carotid artery; IAA, interruption of aortic arch; LSA, left subclavian artery; LAA, left aortic arch; PA, pulmonary artery; RPA, right main pulmonary artery; ALPA, aberrant left pulmonary artery; AA, ascending aorta; CoA, coarctation of aorta; APW, aortopulmonary window; BCT, brachiocephalic trunk.

Fifty-seven children were diagnosed with congenital aortic arch anomalies, including 35 cases of CoA, 6 cases of IAA, 3 cases of main APW, and 13 cases of vascular ring malformation (including 6 cases of a double aortic arch, 5 cases of pulmonary artery sling, and 2 cases of the right aortic arch with aberrant right subclavian artery) (Table 1). Among 35 cases of CoA, 20 cases involved isolated lesions, and 15 cases were complex CoA. Regarding complex CoA, there were 2 cases with ventricular septal defect (VSD), 3 cases with PDA, 3 cases with secundum atrial septal defects (ASDs), 3 cases with VSD and PDA, 3 cases with VSD and secundum ASD, and 1 case with PDA and secundum ASD. Among the 6 cases of IAA, 5 cases were classified as type A, and 1 case was classified as B. Among the 6 cases of the double aortic arch, 2 had secundum ASDs, and the remainder had no intracardiac malformation. Among the 5 cases of pulmonary artery slings, 3 had VSD and secundum ASD, 1 had secundum ASD, and the remaining 2 had no intracardiac malformations (Figure 1).

**Table 1:** Based on the surgical results as the gold standard, results of revolution CT and echocardiography in the diagnosis of congenital aortic arch anomalies.

	Revolution CT				TTE			
	TP	FP	FN	TN	TP	FP	FN	TN
Coarctation of aorta (CoA)	35	0	0	22	25	1	9	22
Interruption of aortic arch (IAA)	6	0	0	51	6	0	0	51
Aortopulmonary window (APW)	3	0	0	54	3	0	0	54
Vascular ring	13	0	0	44	10	0	3	44
Patent ductus arteriosus (PDA)	18	0	1	31	14	2	1	35
Persistent left superior vena cava (PLSVC)	3	0	0	47	2	0	1	47
Secundum atrial septal defect (ASD)	15	2	6	29	20	0	1	29
Ventricular septal defect (VSD)	13	0	2	35	14	0	1	35
Total	106	2	9	313	94	3	16	317

TP, true positive; TN, true negative; FP, false positive; FN, false negative.

All 57 patients with congenital aortic anomalies underwent surgical treatment. The diagnostic sensitivity, specificity, accuracy, positive predictive value, and negative predictive value of CT angiography for congenital aortic arch anomalies (including intracardiac and extracardiac structural abnormalities) were 92.2% (106/115), 99.4% (313/315), 97.4% (419/430), 98.1% (106/108) and 97.2% (313/322), respectively. The diagnostic sensitivity, specificity, accuracy, positive predictive value, and negative predictive value of TTE for congenital aortic arch anomalies (including intracardiac and extracardiac structural abnormalities) were 85.5% (94/101), 99.1% (317/320), 95.6% (411/430), 96.9% (94/97) and 95.2% (317/333), respectively (Table 2). In terms of extracardiac structural malformations, the sensitivity of CTA was 100%, whereas that of TTE was 78.6%. A statistically significant difference between these methods was noted (*P* < 0.001). Regarding intracardiac structural malformations, the sensitivity of CTA was 84.5%, whereas that of TTE was 92.5%. A significant difference was noted between these methods (*P* < 0.001).

**Table 2:** Diagnostic efficacy of CTA and TTE for congenital aortic arch anomalies.

Item		Sensitivity	Specificity	Accuracy	PPV	NPV
CTA	A	100%	100%	100%	100%	100%
	B	84.5%	98.6%	94.6%	96.1%	94.0%
	C	92.2%	99.4%	97.4%	98.1%	97.2%
TTE	A	78.6%	99.4%	94.3%	97.8%	93.4%
	B	92.5%	98.6%	97.0%	96.2%	97.3%
	C	85.5%	99.1%	95.6%	96.9%	95.2%

A, extracardiac structural malformations; B, intracardiac structural malformations; C, intracardiac and extracardiac structural malformations. PPV, positive predictive value; NPV, negative predictive value.

Regarding satisfaction with CTA images, the chief surgeon believed that the CTA images were clear enough to provide adequate preoperative information to confirm the diagnosis with an average score of 3.6. For question 2, the chief surgeon believed that CTA was more effective in determining the surgical approach than TTE, and a statistically significant difference was noted between these methods. The surgeons' responses to the postoperative questionnaire revealed no statistically significant differences between the data provided by CTA and TTE and the degree of intraoperative matching (Table 3). In terms of objective evaluation, the CT values of the ascending aorta, descending aorta, and main pulmonary artery were 358 ± 98.3 (HU), 342 ± 103.6 (HU), and 360 ± 100.3 (HU), respectively. The SD values were 34.8 ± 8.9 (HU), 29.8 ± 7.9 (HU), and 32 ± 8.5 (HU). The SNR values were 10.8 ± 3.9, 11.3 ± 2.9, and 12.9 ± 3.5, respectively. The CT value and SNR value of each major vessel were higher, indicating that the method could obtain good contrast (Table 4).

**Table 3:** Surgeon questionnaire.

Item
1. Do you feel that the image quality of CTA is clear enough to provide information to confirm the diagnosis?					
Answer	Unevaluable	A bit clear	Clear	Very clear	Average
Person (*n*)	1	2	15	39	3.6
2. Do you think TTE/CTA images and measurement data can allow you to decide on the surgical approach for aortic malformations and remind you of intraoperative precautions?					
	TTE		CTA		
Answer	Yes	No	Yes	No	*P* value
Person (*n*)	39	18	53	4	0.004
3. Do you feel that the TTE/CTA measurements match the data you detected during surgery?					
	TTE		CTA		
Answer	Yes	No	Yes	No	*P* value
Person (*n*)	44	13	48	9	0.477
4. How much do you think TTE/CTA has helped you?					
TTE	Useless	A bit useful	Useful	Very useful	*P* value
Person (*n*)	2	11	39	5	0.000
CTA	Useless	A bit useful	Useful	Very useful	
Person (*n*)	1	3	44	9	

**Table 4:** Objective evaluation results of CTA images.

	Ascending aorta	Descending aorta	Main pulmonary artery
CT value (HU)	358 ± 98.3	342 ± 103.6	360 ± 100.3
SD value (HU)	34.8 ± 8.9	29.8 ± 7.9	32 ± 8.5
SNR	10.8 ± 3.9	11.3 ± 2.9	12.9 ± 3.5

## Discussion

Congenital aortic arch anomalies account for 1%–3% of congenital cardiovascular malformations in infants (4). TTE and CT angiography have been widely used as basic cardiac imaging methods for many years. Catheter angiography (CA) is considered the gold standard for diagnosing congenital aortic arch anomalies. However, the patient needs to remain calm for a long time during CA, and complications of endotracheal intubation may occur during this process. Thus, CA is not widely used in clinical practice (5). In addition, it is difficult for CA to accurately identify congenital aortic arch anomalies due to the presence of overlapping large vessels and branches (6). As a routine examination method without any side effects, TTE is simple and convenient to perform and can be used for real-time, dynamic, and multisection imaging. Combined with the color Doppler technique, TTE offers high diagnostic value for intracardiac malformations, especially heart valve lesions, and has become the first choice in the clinic (7). However, the diagnosis of complex CHD has been limited to a certain extent due to the drawbacks of acoustic window images, strong dependence on the operator, and limitations in the assessment of extracardiac large vessel structural abnormalities (8–10). Magnetic resonance imaging (MRI) is a reliable, noninvasive imaging method that can obtain high-quality anatomical and functional images (11, 12). MRI is superior to TTE in the diagnosis of congenital aortic arch malformations in children (11). However, due to the relatively long time of examination and the need for patients to hold their breath, the strict heart rate requirement limits its popularization in clinical application (12). With the development of imaging technology, CT is widely used in the diagnosis of CHD. MSCT has the characteristics of fast scanning speed, large range, high temporal and spatial resolution, noninvasiveness and convenience and has become one of the most potent and valuable methods for cardiovascular noninvasive examination (13). In this study, the sensitivity and accuracy of CTA in the detection and diagnosis of intracardiac malformations were not as good as those of TTE. We found that CTA has a low discrimination rate, especially for those with small secundum ASD and VSD, whereas TTE exhibits a significantly better ability to detect these intracardiac malformations based on the detection of blood flow. In the detection and diagnosis of extracardiac macrovascular malformations, the accuracy of CTA was 100%, whereas that of TTE was 78.9%. This difference might be attributed to the fact that the detection rate of TTE is often limited by the acoustic window and operator's experience. By combining thin-slice axial scanning images with MPR, MIP, VR, and other postprocessing techniques, the Revolution CT system can more clearly and accurately display the details of the inner and outer cardiac structures and reveal the location of the malformation, the degree of cardiovascular stenosis or dilatation, and the relationship between the malformation and the surrounding blood vessels in multiple directions and angles. In this study, 3 cases of aortic arch constriction were missed by TTE. In addition, 6 cases were suspected of aortic arch constriction, but the differential pressure did not meet the diagnostic criteria, resulting in a missed diagnosis. Three cases were diagnosed as misdiagnosis of the vascular ring, including 2 cases of the right aortic arch with aberrant right subclavian artery and 1 case of pulmonary artery sling. CTA exhibits incomparable advantages in the display of the pulmonary artery system and coronary artery system (14). TTE is generally unable to comprehensively observe the whole course of the pulmonary artery, has limited ability to show its course and adjacent spatial relationship, is prone to pulmonary interference and cannot be used evaluate the pulmonary artery system, especially the pulmonary artery branches (15), which might also explain for the missed diagnosis of pulmonary artery sling in our case.

This study used a GE Revolution 512-slice MSCT scanner combined with 70 kV low tube voltage and prospective ECG-triggered technology. The Revolution CT features a 16-cm wide detector, a 0.28-second tube rotation, and SSF algorithm reconstruction technology to significantly improve the time resolution of cardiovascular CT imaging (3, 16, 17). In addition, under the condition of different heart rates or arrhythmias, the whole heart can be scanned by axial scanning within 1 cardiac cycle, which greatly reduces the cardiac and respiratory motion artifacts and greatly improves the diagnostic efficiency and image quality of children with complex CHD. The Revolution CT Intelligent ECG-triggered system intelligently recognizes heart rate and rhythm, matching the optimal scan and reconstruction period accordingly to ensure 1-beat success (18, 19). In addition, ASIR-V reconstruction technology has the advantages of real-time reconstruction and adopts a more advanced system noise model, scanned object model, and physical model, which can further reduce the noise, improve the low density and contrast, and reduce the image artifacts.

In this study, except for 3 cases of poor image quality caused by a lack of cooperation from the children, the images of all the other cases met the needs of diagnosis, and the CTA images were satisfactory from the perspective of objective evaluation indicators. Preoperative images of congenital aortic arch anomalies should allow the surgeon to enter the operating room with complete knowledge of the individual anatomy of the patient and minimize intraoperative exploration or anatomical delineation to develop the best possible surgical plan. In the preoperative questionnaire, surgeons believed that CTA could provide more information on aortic malformations to highlight areas needing attention during surgery. In terms of postoperative questionnaire responses, CTA provides accurate anatomical structure data to surgeons. In addition, we also noted that surgeons believed that although CTA could provide good images, TTE was still indispensable. Surgeons noted that CTA could not be exclusively used to determine the surgical method, and TTE and CTA should be used in combination.

## Limitation

This study was a single center with a small sample size, so it is necessary to further expand the sample size for analysis in future studies. Second, this study only discussed the diagnostic efficacy of the Revolution CT system in congenital aortic arch anomalies, and its application in various complex congenital CHDs should be further explored in the future.

## Conclusion

Wide-detector Revolution CT angiography combined with prospective ECG-triggered CT can be routinely used to assess congenital aortic arch anomalies, providing adequate image quality and high diagnostic accuracy. However, limitations in the identification of intracardiac structural abnormalities are still noted. The advantages of TTE are highlighted in this context.

## Data Availability

The raw data supporting the conclusions of this article will be made available by the authors, without undue reservation.
